# Supplementary material for: Ambient temperature effects on stress-induced hyperthermia in Svalbard ptarmigan
Source: Biol Open. 2019 Jun 10;8(6):bio043497. doi: 10.1242/bio.043497 (PMC6602330; doi:10.1242/bio.043497)
Supplement: Supplementary information [file biolopen-8-043497-s1.pdf]

Table S1. Parameter estimates, likelihood ratios (LRT), and *P*-values (single term deletions), for final models, and *P*-values and test statistics for excluded terms, for models describing body temperature responses to handling in Svalbard ptarmigan in thermoneutrality (0°C) and below thermoneutrality (-20°C) in constant darkness in winter and constant light in spring.

Parameter	Estimate (SE)	LRT	*P*
**Core body temperature (T_c)**			
Response amplitude(°C)			
Final model:			
T_a (-20°C or 0°C)		6.82	**0.009**
T_a = -20°C	0.32 (0.05)		
T_a = 0°C	0.52 (0.04)		
Dropped terms:			
Season (Winter or Spring)		0.00	0.976
Season × T_a		0.28	0.596
Response duration (min)			
Final model:			
T_a (-20°C or 0°C)		14.62	**< 0.001**
T_a = -20°C	5.65 (1.43)		
T_a = 0°C	13.43 (1.11)		
Dropped terms:			
Season (Winter or Spring)		0.62	0.431
Season × T_a		0.90	0.343
Response magnitude (°C)			
Final model:			
T_a (-20°C or 0°C)		5.81	**0.016**
T_a = -20°C	0.17 (0.04)		
T_a = 0°C	0.29 (0.03)		
Dropped terms:			
Season (Winter or Spring)		0.03	0.868
Season × T_a		0.18	0.672
**Back skin temperature (T_{back})**			
Response amplitude(°C)			
Final model:			
-			
Dropped terms:			
T_a (-20°C or 0°C)		2.85	0.092
Season (Winter or Spring)		1.77	0.183
Season × T_a		1.06	0.303
Response duration (min)			
Final model:			
-			
Dropped terms:			
T_a (-20°C or 0°C)		2.74	0.098
Season (Winter or Spring)		0.02	0.881
Season × T_a		1.37	0.242
Response magnitude (°C)			
Final model:			
-			
Dropped terms:			
Season (Winter or Spring)		2.39	0.122
T_a (-20°C or 0°C)		1.60	0.205
Season × T_a		0.58	0.447
**Head skin temperature (T_{head})**			
Response amplitude(°C)			
Final model:			
-			
Dropped terms:			
Season (Winter or Spring)		0.52	0.472
T_a (-20°C or 0°C)		0.58	0.448
Season × T_a		1.40	0.236
Response duration (min)			
Final model:			
-			

Biology Open • Supplementary information

Dropped terms:		
Season (Winter or Spring)	0.02	0.891
T_a (-20°C or 0°C)	0.00	0.946
Season × T_a	0.37	0.543
Response magnitude (°C)		
Final model:		
-		
Dropped terms:		
Season (Winter or Spring)	0.34	0.560
T_a (-20°C or 0°C)	0.07	0.785
Season × T_a	2.21	0.137
